# The Interplay between Central and Peripheral Systems in Feed Intake Regulation in European Seabass (*Dicentrarchus labrax*) Juveniles

**DOI:** 10.3390/ani12233287

**Published:** 2022-11-25

**Authors:** Nicole Martins, Carolina Castro, Aires Oliva-Teles, Helena Peres

**Affiliations:** 1 Departamento de Biologia, Faculdade de Ciências da Universidade do Porto, Rua do Campo Alegre s/n, Edifício FC4, 4169-007 Porto, Portugal; 2 CIIMAR, Centro Interdisciplinar de Investigação Marinha e Ambiental, Universidade do Porto, Terminal de Cruzeiros do Porto de Leixões, Avenida General Norton de Matos s/n 289, 4450-208 Matosinhos, Portugal; 3 FLATLANTIC—Atividades Piscícolas, S.A., Rua do Aceiros s/n, 3070-732 Praia de Mira, Portugal

**Keywords:** fasting, feeding, orexigenic, anorexigenic

## Abstract

**Simple Summary:**

The effect of feeding and feed deprivation on the regulation of feed intake in European seabass was evaluated. European seabass possess feed intake regulation mechanisms similar to those found in mammals and fish. Fasting and feeding conditions induce different responses in feed intake regulation mechanisms in European seabass. The present results are important to identify and understand the mechanisms of appetite regulation of European seabass, and therefore contribute to feeding optimization processes, ensuring the growth and sustainability of the aquaculture industry.

**Abstract:**

The present study aimed to evaluate the effects of feeding or feed deprivation on the orexigenic and anorexigenic responses at the central (whole brain) and peripheral (anterior and posterior intestine, stomach, and liver) system levels in European seabass. For this purpose, a group of fish (208 g) was fed a single meal daily for 8 days (fed group) and another group was feed-deprived for 8 days (unfed group). Compared to the fed group, in the whole brain, feed deprivation did not induce changes in *npy*, *agrp1*, and *cart2* expression, but increased *agrp2* and *pomc1* expression. In the anterior intestine, feed deprivation increased *cck* expression, while in the posterior intestine, the *npy* expression increased and *pyyb* decreased. In the stomach, the *ghr* expression decreased regardless of the feeding status. The hepatic *lep* expression increased in the unfed fish. The present results suggest a feed intake regulation mechanism in European seabass similar to that observed in other teleosts.

## 1. Introduction

Feeding is one of the most important practices in the aquaculture industry, ensuring the good development, growth performance, and welfare of the cultured species. Nowadays, aquaculture mainly relies on the production of fed species, implying the use of aquafeeds [1]. However, aquafeeds represent 50–70% of variable costs [2]. Therefore, understanding the feed intake regulation is of high relevance for the adequate management feeding the profitability of aquaculture.

**Figure 1:**
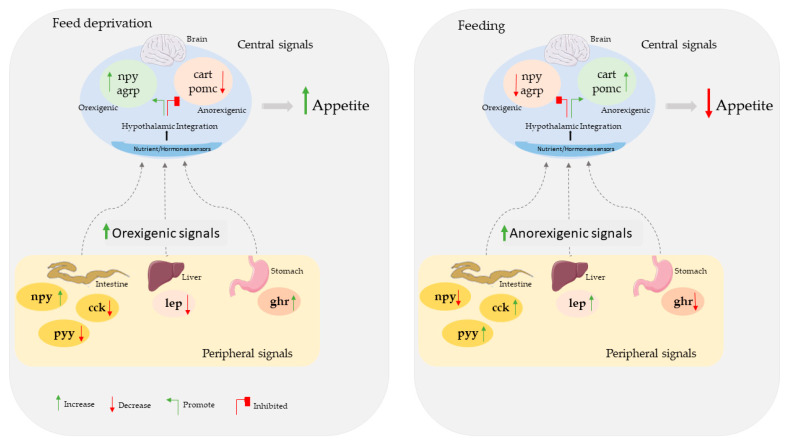
Diagram showing the interaction of peptides and hormones at the central level (brain) and peripheral levels (intestine, liver, and stomach) involved in the regulation of food intake in fish and how they can be affected by feeding deprivation and feeding. agrp: Agouti-related peptide; cart: cocaine- and amphetamine-regulated transcript; cck: cholecystokinin; npy: neuropeptide Y; pomc: proopiomelanocortin; pyy: peptide YY; ghr: ghrelin.

As in other vertebrates, feed intake regulation mechanisms in fish present a complex interaction between the central nervous system, the peripheral systems, and the environment [3,4,5,6]. Furthermore, the animal’s nutritional status provides an important stimulus for the regulation of feed intake mechanisms [5]. Under satiation or fasting, different peripheral peptides and hormones, namely leptin, ghrelin, cholecystokinin, and neuropeptide Y-related peptides, are produced and released into the bloodstream and directed to the hypothalamus. In the hypothalamus, information is integrated and induces changes in the production of orexigenic or anorexigenic neuropeptides that stimulate or suppress appetite [3,6,7] (Figure 1).

The Neuropeptide Y family involves structurally related peptides comprising neuropeptide y (npy), peptide YYa (pyya), peptide YYb (pyyb), and tetrapod pancreatic polypeptide (pp) [8,9]. Npy is considered one of the most potent orexigenic agents in mammals [10]. In fish, npy is found in the central and peripheral systems and has also been described as having potent orexigenic action [4,7]. Npy is regulated by the nutritional status of fish and variations in npy expression were reported in several fish species in response to fasting and refeeding [11,12,13,14,15,16]. For instance, in goldfish (*Carassius auratus*), npy intracerebroventricular injection resulted in an increase in feed intake followed by a decrease in npy mRNA levels after feeding [17]. Additionally, in olive flounder (*Paralichthys olivaceus*), the intraperitoneal injection of npy increased feed intake and growth rate [18].

Pyy is a hormone with a structure similar to that of npy, mostly produced in the intestine L-cells, and has been described as an anorexigenic peptide in mammals [19,20]. In fish, pyy was described to be widely present in both the central and peripheral systems, and to also have anorexigenic functions [9]. Accordingly, in rainbow trout (*Oncorhynchus mykiss*) and Nile tilapia (*Oreochromis niloticus*), the intracerebroventricular injection of pyy decreased feed intake [21,22]. Previous studies also reported that *pyy* expression is affected by feeding or feed deprivation, increasing after a meal and decreasing during fasting, further supporting its anorectic effect in fish [23,24,25,26,27,28].

In mammals, agouti-related protein (agrp) is co-expressed in the hypothalamus along with npy, and also has an orexigenic effect by antagonizing central melanocortin receptors [29]. In fish, two isoforms of the agrp (1 and 2) gene have been identified, and both appear to play a role in the feed intake control by inducing appetite. Accordingly, it was shown that agrp1 and agrp2 mRNA levels increased during fasting, in goldfish (*Carassius auratus*), [30], zebrafish (*Danio renio*) [31], and Atlantic salmon (*Salmo salar*) [32]

Cocaine-and amphetamine-regulated transcript (cart) is a peptide with neurotransmitter and neuroendocrine functions with numerous key roles in pituitary hormone secretion, and energy metabolism, acting as an anorexigenic agent across the vertebrate phyla [4,6,7,33]. In goldfish, it was shown that intracerebroventricular cart injections decreased feed intake and growth rates [34]. Additionally, fasting decreased brain cart expression in several fish species, further supporting its anorexigenic action [11,14,35,36,37].

Proopiomelanocortin (pomc) is a precursor peptide that is post-transcriptionally processed into melanocortins, including melanocyte-stimulating hormones (a-, b- and c-MSH) and adrenocorticotropic hormone (ACTH). In mammals, pomc neurons produce α-MSH and cart, both inducing a decrease in feed intake [38]. As in mammals, fish hypothalamus co-expresses pomc and cart. However, the pomc role in feed intake regulation still needs to be clarified. For instance, in Atlantic halibut (*Hippoglossus hippoglossus*), pomc expression was higher half an hour after feeding but no differences were observed in the following hours [25]. On the contrary, in olive flounder (*Paralichthys olivaceus*), fasting increased pomc expression [39].

In mammals, cholecystokinin (cck) produced and released by the gastrointestinal tract endocrine cells acts as a satiety signal, reducing feed intake. In fish, cck is widely expressed both in the brain and gastrointestinal tract, and it was also shown to have an anorexigenic action in winter skate (*Raja ocellata*) [40], blunt snout bream (*Megalobrama amblycephala*) [41], white sea bream (*Diplodus sargus*) [42], channel catfish (*Ictalurus punctatus*) [13], and Ya fish (*Schizothorax prenanti*) [16].

In fish, as in mammals, ghrelin (ghr) is mainly synthesized in the stomach and is considered the most potent orexigenic hormone produced in the gastrointestinal tract [43]. Supporting its orexigenic action, it was shown that ghr increased after feed intake in goldfish [44,45], brown trout (*Salmo truta*) [46], cavefish (*Astyanax fasciatus mexicanus*) [47], and blunt snout bream [41]. However, in rainbow trout, contradictory results were obtained, as it was suggested that ghr might have orexigenic [48] and anorexigenic [49] functions.

Leptin (lep) is an anorexigenic hormone with several physiological functions in energy balance, fat deposition, and feed intake regulation, and in mammals, it is synthesized in the adipose tissue [50]. In contrast, in fish, even though adipose tissues also produce lep [51], the main lep producer is the liver. As in mammals, lep was also described as having a potent anorexigenic function in fish. Accordingly, it was shown that fasting led to a decrease in lep mRNA levels in striped bass (*Morone saxatilis*) [52], common carp (*Cyprinus carpio*) [53], and Ya fish [16].

European seabass (*Dicentrarchus labrax*) is a well-established species in European aquaculture, with a production of 84.430 tonnes in 2019 [54]. Thus, optimizing feeding without compromising the growth performance and health of European seabass is of great interest to the aquaculture industry. However, feeding optimization implies a deep knowledge of feeding control mechanisms and how feeding status affects feed intake. Thus, this study aimed to contribute to the understanding of the response mechanisms involved in feed intake regulation in European seabass juveniles.

## 2. Materials and Methods

### 2.1. Experimental Conditions

The trial was carried out at the Marine Zoological Station, Porto University, in a thermoregulated recirculation water system equipped with 8 cylindrical fiberglass tanks of 100 L capacity and supplied with a continuous seawater flow (3 L min^−1^). The trial was performed by accredited scientists according to FELASA category C recommendations and approved by the CIIMAR ethical committee for Managing Animal Welfare (ORBEA), in conformity with the European Union directive 2010/63/EU and the Portuguese Law (DL 113/2013).

European seabass was obtained from a commercial farm (Acuinuga S.L., Bertamiráns, Ames, Spain) and submitted to a quarantine period of one month before the trial. Then, fish with an average body weight of 208.0 ± 0.3 g were randomly distributed to 8 tanks (6 fish/tank) and two groups were randomly established as fed and unfed (four tanks per group). Both groups were fed once daily until apparent satiation with a commercial diet (Aquasoja Sustainable Feed-Standart Orange 4 MEO4; Sorgal, Ovar, Portugal) for 16 days, and then, the fed group continued to be fed for 8 days as above while the unfed group was kept without feeding during the while period.

During the trial, the water temperature was maintained at 23 ± 1 °C, salinity at 35 ± 1‰, dissolved oxygen was kept near saturation, ammonia and nitrites levels were kept around zero mg L^−1^, and a photoperiod of 12 h light:12 h dark was adopted.

At the end of the trial, both groups were sampled before (0 h) and at 3, 9, and 12 h hours after the feeding time. To prevent stress due to manipulation, a different tank of each group was sampled at each sampling time. The blood of six fish per treatment and sampling time was collected from the caudal vein using a heparinized syringe, placed in heparinized tubes, and centrifuged at 6800× *g* for 10 min at room temperature. The aliquots of the resultant plasma were stored at −20 °C for further analysis. After blood collection, the fish were euthanized with a sharp blow to the head and whole brain, and samples of the posterior and anterior intestine, stomach, and liver were collected, kept in RNA later (1:10) at 4 °C for 24 h, and then stored at −80 °C until RNA extraction.

### 2.2. Plasma Metabolites 

Commercial kits from Spinreact, S.A. (Gerona, Spain) were used for plasma glucose (Kit cod. 1001191), triglycerides (Kit cod. 1001312), cholesterol (Kit cod. 1001092), and phospholipids (Kit cod. 1001140) analysis. All plasmatic parameters were analyzed using colorimetric reactions and absorbance was read in a Multiskan GO microplate reader (Model5111 9200; Thermo Scientific, Nanjing, China).

### 2.3. Gene Expression 

The RNA of each tissue was isolated through homogenization in a Precellys evolution apparatus (Bertin Instruments, Montigny-le-Bretonneux, France) with TRIzol reagent (Direct-zolTM RNA Miniprep, Zymo Research) and following manufacturer recommendations. The RNA quality was assessed by electrophoresis on 1% agarose gel and the RNA quantity was checked by spectrophotometry (µDrop™ plate, ThermoScientific, Waltham, VA, USA). RNA concentration was adjusted to 0.5 µg/8 µL H_2_O for complementary DNA synthesis, utilizing the NZY First-Strand cDNA Synthesis Kit (NZYTech. MB12502, Lisbon, Portugal).

Gene expression was determined by real-time quantitative PCR analysis (CFX Connect™ Real-Time System, Bio-Rad, Hercules, CA, USA). The analysis mixture contains 0.4 µL diluted cDNA (1:1), 0.2 µL of each primer (10 µΜ), 5 µL SsoAdvanced Universal SYBR^®^ Green supermix (Bio-Rad), and 4.2 µL DNase/RNASE/Protease-free water, in a total volume of 10 µL. The sequences of primers used are presented in Table 1. The pair of primers’ quality was analyzed with the Beacon Designer Program. Primer efficiency was validated with serial two-fold dilutions of cDNA and determined from the slope of the regression line of the quantification cycle (Ct) versus the relative concentration of cDNA [55]. Primer efficiency among 90 and 110% (slope −3.5 and 3.1, r^2^ = 0.99) were accepted. For agrp1, agrp2, npy, pomc1, cart2, cck, ghr, and lep primers pairs, the annealing temperature was determined through a temperature gradient. The reaction was initiated with incubation at 95 °C for 30 s for hot-start iTaqΤΜ DNA polymerase activation. A total of forty PCR cycles were performed, each consisting of heating at 95 °C for 15 s for denaturing, and 30 s for annealing and extension. The annealing temperature of each primer pair is presented in Table 1. Melting curves were systematically monotonized (60 °C temperature 0.5 °C 10 s^−1^ from 60 to 95 °C). Each PCR run included triplicates of reverse transcription for each sample and negative controls (reverse transcriptase-free samples, RNA-free samples). To normalize the results, previously validated reference genes, namely elongation factor 1α (ef1α) and 18S ribosomal RNA (18s), were used. The expression levels are given as the mean normalized values ± standard error (SE) corresponding to the ratio between copy numbers of the target gene transcripts and the geometric mean of copy numbers of the reference genes [56].

### 2.4. Statistical Analysis

All data were tested for normality and homogeneity of variances by the Shapiro–Wilk and Levene tests, respectively, and, when necessary, were log-transformed. Statistical analysis was performed by two-way ANOVA with feeding status (fed and unfed) and sampling times (0, 3, 9, and 12 h) as the main factors. When significant interaction between the main factors was observed, one-way ANOVA was used for each factor. Significant differences between the means were determined by Tukey’s multiple range test. The level of significance used was *p* < 0.05 for all statistical tests. All the statistical analysis was performed using the SPSS version 27 software package for Windows (IBM^®^ SPSS^®^ Statistics, Armonk, NY, USA).

## 3. Results

**Figure 2:**
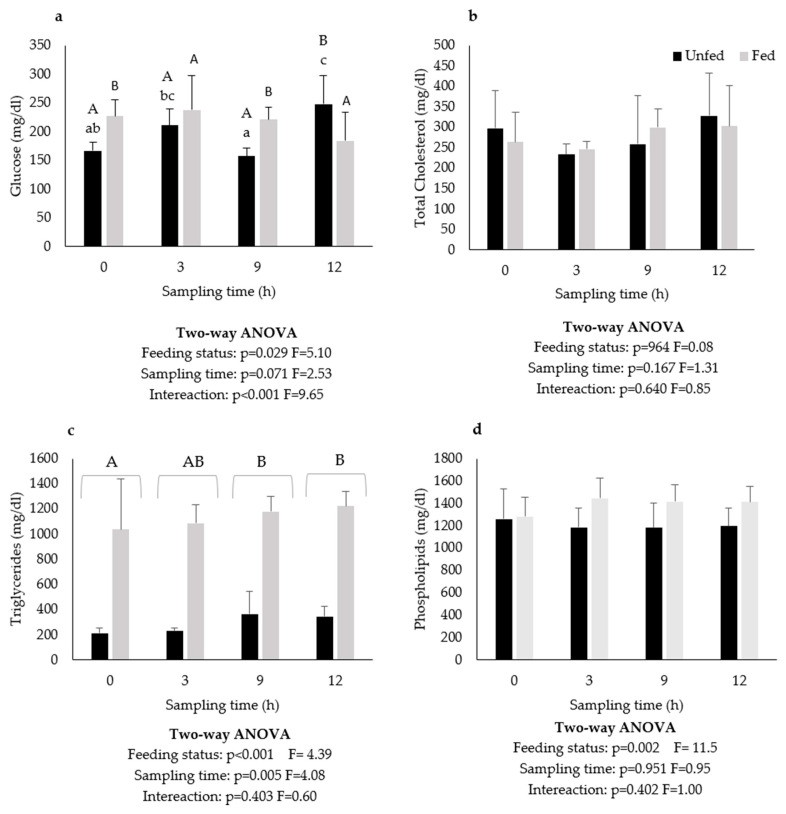
Plasma glucose (**a**), total cholesterol (**b**), triglyceride (**c**), and phospholipid (**d**) levels of European seabass juveniles after 8 days pf feed deprivation (unfed) or continuous feeding (fed). Values are presented as means (n = 6) and standard error (SE). Small letters denote significant differences in sampling times regardless of feeding conditions and capital letters denote significant differences in sampling times between feeding conditions.

Plasmatic glucose levels were higher in the fed than in the unfed group, except at 12 h where the opposite was observed. No statistically significant differences in plasma glucose were observed with time in the fed group, while in the unfed group the highest glucose levels were observed at 3 and 12 h, and the lowest at 9 h (Figure 2a). Plasma cholesterol levels were not affected by feeding status or postprandial delay (Figure 2b). Plasma triglyceride levels were much higher in the fed (1000–1200 mg dL^−1^) than in the unfed (200–300 mg dL^−1^) group and were higher at 9 and 12 h than at the other sampling times, regardless of feeding status (Figure 2c). Plasma phospholipid levels were also higher in the fed (1400 mg dL^−1^) than in the unfed group (1200 mg dL^−1^) and no differences between sampling times were observed, regardless of the feeding status (Figure 2d).

**Figure 3:**
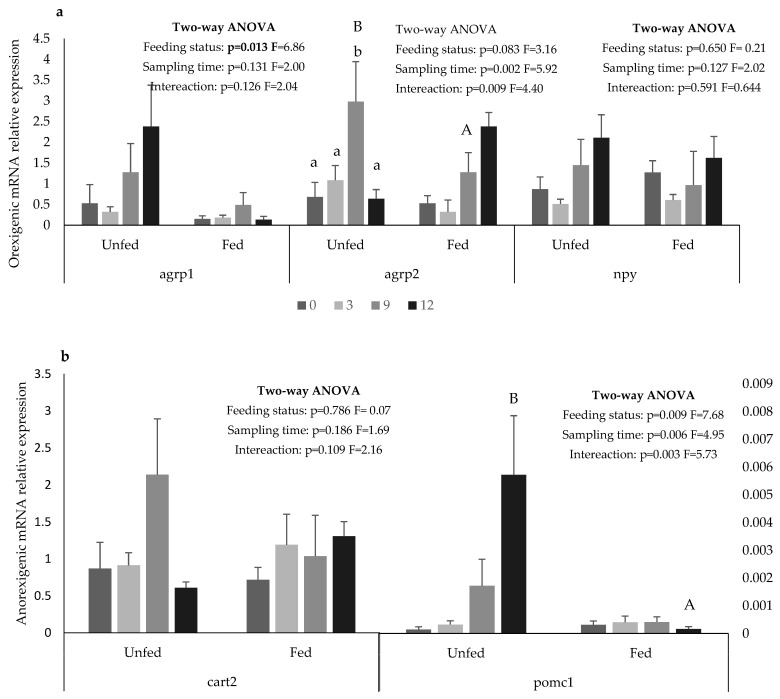
Orexigenic (**a**) and anorexigenic (**b**) expressions in the central system of European seabass juveniles after 8 days of feed deprivation (unfed) or continuous feeding (fed). Data were normalized with two housekeeping genes, ribosomal 18S (18S) and α-elongation factor (ef1), according to [56]. Values are presented as means and standard error (SE). Small letters denote significant differences in sampling time under the same feeding conditions and capital letters denote significant differences in sampling times regardless of the feeding conditions. py: neuropeptide Y; agrp: Agouti-related protein; cart: cocaine-and amphetamine-regulated transcript; pomc: proopiomelanocortin.

In the brain, *npy* and *cart2* expression were not affected by the feeding status or sampling times (Figure 3a,b, respectively). Differently, the *agrp1* expression was 1-fold higher in the unfed than in the fed group and was not affected by sampling times (Figure 3a). The expression of *agrp2* was not affected by sampling time except in the unfed group, where it peaked at 9 h (Figure 3a). The expression of *pomc1* was not affected with time in the fed group, while in the unfed group, it increased from 9 h onwards, and at 12 h, it was 30-fold higher than in the fed group (Figure 3b). The Cck expression was not detected in the brain, either in the unfed or the fed fish.

**Figure 4:**
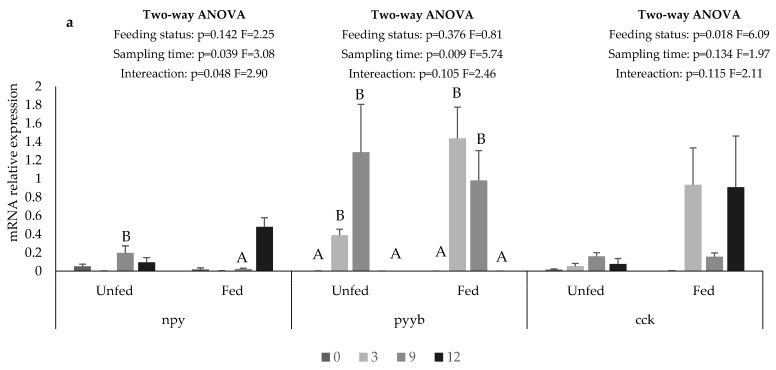

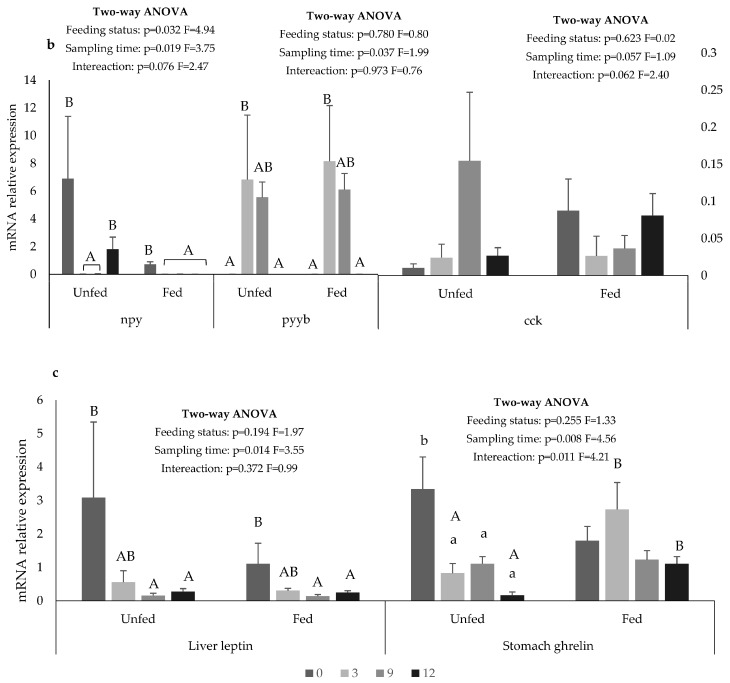
mRNA relative expression of main hormones and peptides in the peripheral systems anterior intestine (**a**), posterior intestine (**b**), liver and stomach (**c**) of European seabass days after feed deprivation (unfed) or continuous feeding (fed). Data were normalized with two housekeeping genes, ribosomal 18S (18S) and α-elongation factor (ef1), according to [56]. Values are presented as means and standard error (SE). Small letters denote significant differences in sampling time under the same feeding conditions and capital letters denote significant differences in sampling times regardless of feeding conditions. npy: neuropeptide Y; pyyb: peptide YYb; cck: cholecystokinin; lep: leptin; ghr: ghrelin.

In the anterior intestine, *npy* expression at 9 h it was 9-fold higher in the unfed than in the fed group, while at 12 h *npy* expression, it was 5-fold higher in the fed group than the unfed group, although differences between groups were not statistically significant (Figure 4a). The expression of *pyyb* was not affected by the feeding status and was higher at 3 and 9 h than at the other sampling times (Figure 4a). In contrast, the *cck* expression was not affected by the sampling time but was higher in the fed than in the unfed group (Figure 4a).

In the posterior intestine, the *npy* expression was lower in the fed than in the unfed group and, regardless of feeding status, it was higher at 0 h and 12 h than at the other sampling times (Figure 4b). The *pyyb* expression was not affected by the feeding status, but it was higher at 3 and 9 h than at the other sampling times (Figure 4b). The *cck* expression was not affected by feeding status or sampling times (Figure 4b).

In the stomach, the *grh* expression decreased with time in the unfed group while in the fed group it was not affected by the sampling time, and at 3 and 12 h, was 2-fold higher than in the unfed group (Figure 4c). Liver *lep* expression was not affected by feeding status and decreased with time from 0 to 9 in both groups (Figure 4c).

## 4. Discussion

Previous studies in European seabass have already reported the presence of some peptides and hormones that are known to be involved in feed intake regulation. However, their specific role in feed intake regulation in this species was not confirmed [57,58,59,60,61,62]. Thus, the present study aimed to elucidate the anorexigenic or orexigenic responses of some of these peptides and hormones in the central and peripheral systems of European seabass. As plasmatic biochemistry parameters are affected by feeding conditions, in this study, we also assessed the specific plasmatic metabolites that are known to be good indicators of the nutritional condition of European seabass [63].

In the present study, plasma glucose levels after 8 days of feed deprivation were circa 30% lower than that of fed fish. This is in agreement with the results of Pérez-Jiménez et al. (2007), also in this species, which reported a significant drop in plasma glucose on the first day of feed deprivation, with values remaining stable thereafter for at least 8 days. In contrast, Peres et al. (2014) reported that glucose levels were maintained for starvation period of at least 7 days. In the present study, no statistical differences in plasma glucose were noticed with time in the fed group. However, in absolute values, plasma glucose levels were higher at 3 h post-feeding (hpf) and decreased thereafter. Similarly, in a glucose tolerance test with European seabass, Peres et al. (1999) also observed that plasma glucose levels peaked at 3–6 h, decreasing thereafter.

In the present study, plasma triglyceride levels were nearly 80% lower in unfed fish compared to fed fish, while total cholesterol and phospholipids were not affected by feeding status. Peres et al. (2014) also reported a significant decrease in plasma triglyceride levels after 7 days of starvation while the total cholesterol levels were unaffected. Similarly, Pérez-Jiménez et al. (2007) observed a decrease in plasmatic triglycerides levels after 9 days of starvation. However, in contrast with the present study and that of Peres et al. (2014), these authors also reported a reduction in cholesterol levels after 9 days of starvation.

Npy is a potent orexigenic peptide in fish, mainly expressed in the brain and intestine [7,9,64]. *Npy* expression is known to vary with diet composition, meal frequency, and ration size, and circadian rhythm and feed deprivation are major inducers of npy expression [9,65,66]. On the contrary, during feeding, npy discharge usually occurs near feeding time and then it progressively decreases as feed ingestion proceeds [9]. In the present study, *npy* expression in the brain was similar in fed and unfed fish, suggesting that *npy* may not have an orexigenic role in European seabass. These results agree with those observed in Atlantic halibut [25], Atlantic salmon [35], and Atlantic cod (*Gadus morhua*) [11] but are contradictory to those observed in other fish species. For instance, in blunt snout bream [41], yellowtail [15], Nile tilapia [22], and channel catfish [13] *npy* expression in the brain increased through fasting, and decreased during feeding. Additionally, on the contrary to present results, other studies have reported a pre and postprandial variation of *npy* expression in response to a meal. For instance, in goldfish, *npy* expression increased 1–3 h before the meal and decreased 1–3 hpf [17]. Similarly, in Brazilian flounder (*Paralichthys orbignyanus*), the *npy* expression was elevated at mealtime and reduced at 2 hpf [67], and in fed Ya-fish, the *npy* expression decreased at 0.5, 1.5, 3, and 9 hpf [16].

It is known that the *npy* expression may differ in the different regions of the brain and analyzing the expression in the whole brain may not be sensitive sufficiently sensitive to detect differences in expression in the different brain regions. For instance, in goldfish, [17] observed a higher *npy* expression in telencephalon-preoptic than in the hypothalamus. In contrast, in winter skate, hypothalamic *npy* expression was not affected by feeding or fasting, while in the telencephalon, it was higher in fasted animals [14]. In Atlantic cod, the *npy* expression was higher in the telencephalon than in the hypothalamus and optic tectum, regardless of feeding status [11]. Further studies in European seabass should analyze the *npy* expression in different regions of the brain to further elucidate the role of npy in feed intake regulation.

In mammals, npy acts as a brain–gut peptide influencing gastrointestinal processes at the peripheral system level [20], and in fish, it was shown that it inhibits the contractile activity of the stomach in elasmobranchs [68,69,70], and induces vasorelaxation and intestinal contractions in Atlantic cod [71]. In the present study, fasting increases *npy* expression in anterior and posterior intestines, suggesting that, as in mammals, npy may act as a brain–gut peptide in European seabass. This agrees with what was observed in other fish species, including Atlantic cod [11], grass carp (*Ctenopharyngodon idellus)*, [72] winter skate [14], winter flounder (*Pseudopleuronectes americanus)* [40], and yellowtail [15].

Peptide YY (*pyy*) and peptide Y (*py*) are npy-related peptides that have been described as important gut peptides in feed intake regulation and energy homeostasis [10]. There is, however, some controversy regarding the exact function and the evolutionary relationship of these peptides with the peptides of the npy-family. Previously, it was suggested that European seabass has orthologues of npy and pyy as well as py [73]. Py seems to be a pancreatic peptide present in fish instead of the pancreatic polypeptide (pp) that is found in mammals. Cerdá-Reverter et al. (2000) proposed that py arose from a copy of pyy after gene duplication and besides the pancreas, it is expressed in the brain and intestine, which are the main feed intake control areas. Upon reevaluating the phylogenetic evolution of the neuropeptide Y family, Sundström et al. (2008) concluded that py emerged as a duplicate of the *pyy* gene and should hence be called pyyb. The *pyy* gene (either *pyya* or *pyyb*, according to Sundström et al. (2008) was identified in several fish species, including Atlantic salmon [32], goldfish [23], Ya-fish [24], zebrafish, and pufferfish [74], and Nile tilapia [22], and was shown to have an anorexigenic function. In the present study, the *pyyb* expression was not affected by feeding status. However, it increased at 3hpf and then decreased with sampling time both in the anterior and posterior intestines, suggesting that it may have an anorexigenic function, as in other fish species. Further studies are, however, necessary to better elucidate the role of pyy in the feed-intake regulation in European seabass.

The neuropeptides agrp1 and agrp2 are involved in energy regulation, and their orexigenic role in feed intake regulation was confirmed in several fish species, including Atlantic salmon [35,75], zebrafish [31,76], goldfish [77], Ya fish [16], and Arctic Charr (*Salvelinus alpinus*) [78]. Both agrp isoforms were previously confirmed in European seabass and were shown to be mainly expressed in the brain, ovary, posterior kidney, pineal organ, and ventral skin [58]. It was also observed that *agrp1* expression increased during a long-term fasting period (8–29 days), while *agrp2* decreased after 15 days of fasting [58]. This suggests that *agrp1* and *agrp2* may have different mechanisms of action in the feed intake regulation of European seabass. Accordingly, in the present study, *agrp1* expression was affected by the feeding status being higher in the unfed fish, while *agrp2* expression was similar in both groups, except at 9 h, at which it peaked in the unfed fish. Further studies are needed to better understand the specific role of agrp1 and agrp2 in the feed intake regulation of European seabass.

The cart gene is strongly conserved within vertebrates, and conserves several physiological functions in different species [33]. In fish, several cart isoforms have been described, possibly due to the genome duplication that fish experienced [4]. For instance, seven *cart* isoforms were identified in Senegalese sole (*Solea senegalensis*). However, only three isoforms (*cart1a*, *cart2a,* and *cart4)* have been shown to respond to feeding, being up-regulated in the post-feeding period [37]. To date, only one cart isoform, *cart2* was identified in European seabass [79]. In the present study, *cart2* expression was similar in fed and unfed European seabass. The absence of *cart2* response to feeding could be related to the isoform evaluated, which may not be the one involved in feed intake regulation. Accordingly, Bonacic et al. (2015) identified two *cart2* isoforms in Senegalese sole, but only *cart2a* responded to feeding. Thus, further studies are necessary to establish the possible existence of cart isoforms in European seabass.

Although the presence of several cart isoforms is well established in fish, in some species, the existence of only one cart isoform was reported. In such cases, and similarly to what was observed in the present study, there was no modulation in cart expression in fed or unfed fish. [14,25].

In European seabass, only one functional *pomc* isoform (pomc1) was identified, and it was mainly expressed in the central system [59]. In the present study, *pomc1* expression in the whole brain was very low, and almost undetected in the fed European seabass. In the unfed group, an increase in *pomc1* expression was observed between 9 and 12 h, suggesting an orexigenic role of *pomc1* in feed intake regulation in European seabass. In fish, the role of *pomc* in feed intake regulation is still largely unexplored and seems to be dependent on the *pomc* isoform, fish species, and fish nutritional status. For instance, in olive flounder, three *pomc* isoforms were identified, while the *pomc1* and *pomc3* expressions were not affected by feeding status, the *pomc2* expression increased during fasting, suggesting that it has an orexigenic role in this species [39]. In Atlantic salmon, four functional *pomc* isoforms were identified (*pomca1/a2/a2s/b*), but only *pomca1* expression was affected by feeding, increasing at 3 hpf, and indicating that it had an anorexigenic role [80]. In rainbow trout, 14 days of feed deprivation did not affect hypothalamic *pomca1, pomca2, pomca2s,* or *pomcb* expression, but a 50% reduction in *pomca1* expression was observed after 28 days of fasting [81].

Cck is an important gut hormone, previously described as having a potent anorexigenic function in European seabass [82]. The authors showed that the oral administration of cck decreased the appetite of European seabass. Accordingly, in the present study, in the fed group, an increase in cck expression was observed at 3 and 12 hpf, while in the unfed group, the cck expression was lower and similar along the sampling times. In contrast, in the posterior intestine, no effects of feeding or fasting were observed in the *cck* expression. These results further support an anorexigenic action of cck in European seabass and are in line with the results observed in other fish species, such as yellowtail [83,84], blunt snout bream [41], cunner [85], zebrafish [86], winter flounder [40], Atlantic salmon [80], and channel catfish [13].

In fish, the role of *grh* in feed-intake control is still controversial. For example, an orexigenic action of *ghr* was reported in grass carp [87], blunt snout bream [41], zebrafish [88], goldfish [44], while an anorexigenic function was reported in Atlantic salmon [89] and rainbow trout [49]. In European seabass, ghr was reported as a potent orexigenic hormone, being up-regulated under starvation and downregulated during refeeding [61]. In the present study, in the fed group, *ghr* expression was higher—up to 3 h and 12 h after the meal. On the contrary, in the unfed group, *ghr* expression was higher at 0 h and then decreased until null expression was observed at 12 h. This supports the orexigenic action of *ghr* in European seabass, although it also indicates that even after 8 days of fasting, fish still seem to respond to the previously circadian feeding conditioning.

As in mammals, lep is involved in the regulation of energy balance in fish [90], interacting with different peptides and hormones, such as agrp, npy, and pomc in a feedback manner, decreasing the expression of orexigenic genes and up-regulating anorexigenic genes [51,53]. Previous studies reported an anorexigenic action of lep in fish [91]. In European seabass, lep appears to act differently. According to Gambardella et al. (2012), *lep* expression increased during fasting and decreased after refeeding, and in the present study, no significant differences in *lep* expression were observed between the fed and unfed fish. Further studies are needed to confirm the role of lep in the feed intake regulation of European seabass.

## 5. Conclusions

In conclusion, the present study shows that in European seabass, feeding did not induce significant changes in orexigenic and anorexigenic peptides and hormones at the central and peripheral levels. Feeding deprivation increased the *agrp2* expression in whole-brain and *npy* in posterior intestine suggesting an orexigenic action. Further studies are necessary to further understand the role of the different neuropeptides and hormones in the feed intake regulation of European seabass and the specific mechanisms of action.

## Figures and Tables

**Table 1:** Sequences of primers used for real-time quantitative PCR determination of several genes involved in the feed-intake regulation mechanism in European seabass (*Dicentrarchus labrax*) ^1^.

Gene	Sequence (5′-3′)	Ta (°C)	Efficiency	Accession Number
Central				
agrp1	F: TCTGCTGCGTCTCTCTTCTT	62.0	2.01	HE660086
	R: TCTCTCCAGGTCAGACAGGT			
agrp2	F: GGGCAGAGGACACAAAGAAA	62.0	2.01	HE660087
	R: TGTGACTTTCCTGTGGTGGA			
cart2	F: CCGAACCTGACCAGCGAGAA	61.0	1.92	MZ441181
	R: GCTCCCCGACATCACACGTT			
npy	F: ACTCAGCCCTGAGACACTACA	50.8	2.01	AJ005378
	R: ACTGTGGAAGCTGGTCTGTG			
pomc1	F: TCCCTGTTCTCTTCCTCCTC	57.6	2.01	AY691808
	R: CTCTGCCGCTATAATCTCGC			
Peripheral				
cck	F: TTCTCCTCCCAGCTCTTTGA	60.0	1.92	DLA_LG9_001730
	R: GGAGTCTGCATCCTCCTCAG			
ghr	F: TTGCTGGTGGTTCTGTTGTG	61.0	1.95	DQ665912
	R: TTGCCTCTGCTCTGAGGTTT			
lep	F: TCAGTCAGTGGAGGCAAGAG	60.0	1.92	KJ934254
	R: TTTCAGCAGCAGATTGAGGA			
pyyb	F: GGTGTGTTTGAGCAGCTTCG	60.0	1.92	AJ005380
	R: CAGCGTCTTGGCTTGAATCG			
Housekeeping				
ef1α	F: GCTTCGAGGAAATCACCAAG	60.0	1.97	AJ866727
	R: CAACCTTCCATCCCTTGAAC			
18s	F: GGAGGTTCGAAGACGATCAG	63.0	2.01	AY831388.1
	R: GAGGTTTCCCGTGTTGAGTC			

^1^ agrp1, agouti-related peptide 1; agrp2, agouti-related peptide 2; cart2, cocaine-and-amphetamine-regulated transcript; npy, neuropeptide Y; pomc1, proopiomelanocortin 1; cck, cholecystokinin; ghr, ghrelin; lep, leptin; py, peptide Y; ef1α, elongation factor 1α; 18s, 18S ribosomal rRNA; Ta, annealing temperature.

## Data Availability

Not Applicable.
